# Clinical Insight Into a Rare Anatomical Variation: An Autobiographical Case Report of a Supernumerary Extraocular Muscle (SEOM) in a Patient With Hyperthyroidism

**DOI:** 10.7759/cureus.46887

**Published:** 2023-10-12

**Authors:** Vishel Soundarajan, Gunavathy Nandakumal, Preetvinder Singh Dheer Singh

**Affiliations:** 1 Ophthalmology, Hospital Raja Permaisuri Bainun, Ipoh, MYS; 2 Radiology, Hospital Raja Permaisuri Bainun, Ipoh, MYS

**Keywords:** thyroid eye disease (ted), ocular anomaly, optic nerve compression, restrictive strabismus, lid retraction, hyperthyroidism, supernumerary extra ocular muscle

## Abstract

In this case report, we present the rare occurrence of supernumerary extraocular muscles (SEOM) in a 35-year-old male with hyperthyroidism. SEOMs are unusual anatomical variations involving extraocular muscles that deviate from the typical muscle arrangement in the eye. While SEOMs are rare, they can have diverse clinical manifestations, including restrictive strabismus and lid abnormalities. In this case, the patient displayed right-sided lid retraction and an asymmetrical palpebral aperture, which raised concerns about a potential association with thyroid eye disease. However, imaging revealed that the SEOM was anatomically connected to the superior rectus muscle, possibly contributing to the observed lid retraction. Understanding the complexities of SEOM and its potential interactions with conditions like thyroid ophthalmopathy is crucial for accurate diagnosis and management. Further research is needed to fully comprehend the development and clinical impact of SEOMs due to their rarity and limited knowledge in the medical literature.

## Introduction

Supernumerary extraocular muscles (SEOMs) have been mentioned in historical literature using various terminologies such as orbital bands, accessory EOMs, supernumerary bands, and anomalous EOMs. The earliest reported case of SEOM was by Nussbaum M in 1893, describing an abnormal tissue arising from the tendinous origin with three anterior heads attaching to the superior, lateral, and inferior rectus muscles [1-2]. Knowledge regarding SEOM is limited due to its rarity and low incidence [2]. However, Khitri et al. conducted a prospective study utilizing high-resolution orbital MRI in 118 normal subjects without strabismus and 453 strabismic subjects, finding prevalence rates of anomalous extraocular muscle bands to be 0.8% and 2.4%, respectively, with no statistical significance between the groups [3].

Several theories in the literature propose the development of these anomalous muscles. One hypothesis suggested by Whitnall in 1911 proposes that SEOMs may represent atavistic retractor bulbi muscles, typically found in reptiles, amphibians, and some ruminants but not in humans [4,5]. Another theory suggests an early disturbance in the development of the superior and inferior mesenchymal/mesectodermal complexes with incomplete division of some of its parts [6]. Khitri et al. also supported this theory, suggesting that the anomalous EOM bands may represent atrophied residual orbital remnants of aberrant muscular tissue that either were never innervated or lost innervation during development [3]. Common presentations of SEOMS are related to restrictive strabismus. However, in this report, we present a case of SEOM in a patient with hyperthyroidism with a critical anatomical location that could have early, potentially severe visual implications if thyroid ophthalmopathy were to occur.

## Case presentation

A 35-year-old Indian male presented with a one-day history of asymmetrical lid position, with the right eye appearing larger than the left in a photograph. The patient had no known comorbidities and denied any previous awareness of the asymmetry. Upon review of old photographs, it was discovered that the asymmetry had been present in a photo taken one year ago. The patient also reported experiencing dry eye over the right eye, leading to intermittent blurring of vision over the past five years. No other symptoms were noted.

**Figure 1:**
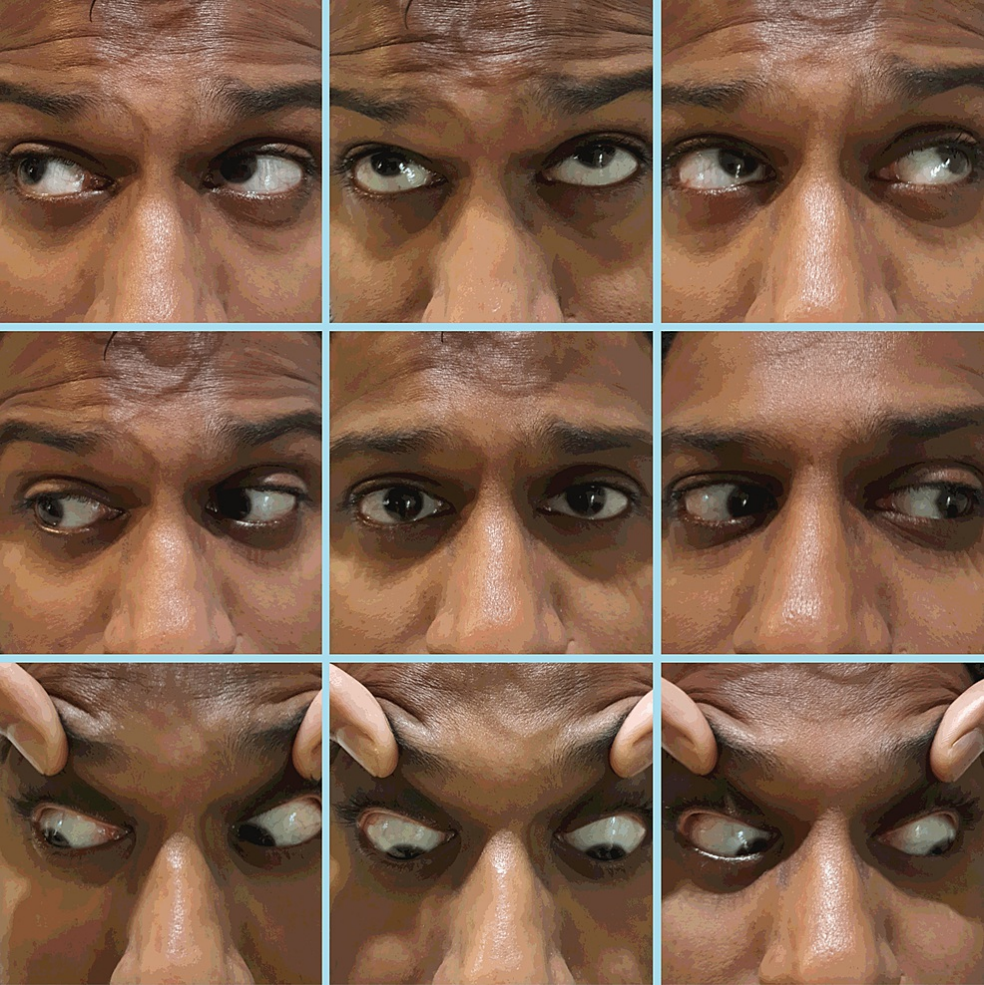
Nine-gaze photo showing lid retraction of the right eye in the primary position and no ocular restrictions in other gazes

The patient's visual acuity was measured as 6/9 in both eyes, and a negative relative afferent pupillary defect was detected. There were no signs of ocular motility restriction or strabismus. Clinically, there was suspicion of proptosis; however, assessment with an exophthalmometer did not reveal any evidence of it. Lid examination revealed a retracted right upper lid resting over the limbus but there were no motility restrictions of the globe (Figure 1). Palpebral aperture measurements were 12 and 9 millimeters in the right and left eyes, respectively.

Further examination of the anterior segment of both eyes was normal, and the intraocular pressure in primary gaze and upgaze was within the normal range (10 mmHg and 12 mmHg, respectively). However, the fundus examination showed an asymmetrical cup-disc ratio of 0.8 in the right eye and 0.4 in the left eye, with no evidence of glaucomatous damage. Humphrey's visual field examination did not reveal any abnormalities. Optical coherence tomography of the optic nerve head was also performed, which did not reveal any significant abnormality. There were no other findings suggestive of Graves disease.

Given the suspicion of thyroid eye disease, the patient was referred to an endocrinologist. The thyroid function tests indicated T3 toxicosis, as evidenced by an elevated T3 level and suppressed thyroid-stimulating hormone (TSH). Specifically, the results were as follows: TSH 0.31 mIU/L (reference range: 0.35-4.94), free T4 15.0 pmol/L (reference range: 9-19), and free T3 6.2 pmol/L (reference range: 3.5-6.0). As a result, the patient was started on systemic carbimazole 10 mg once daily to manage the thyroid dysfunction. Antibody screening was also performed, including anti-TSH receptor antibody, thyroid-stimulating immunoglobulin (TSI), and anti-thyroperoxidase antibody, which were negative.

**Figure 2:**
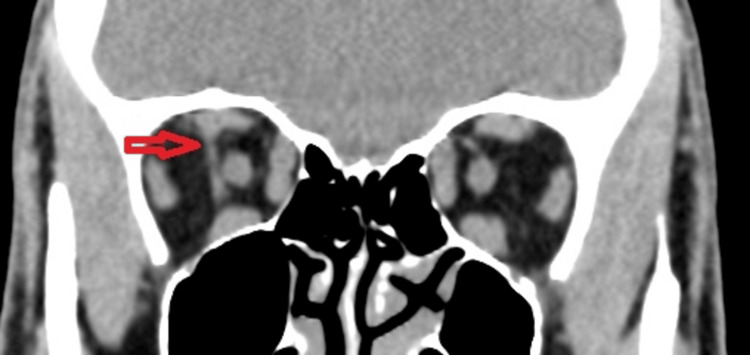
Coronal view of a plain computed tomography showing the orbit of the patient with an elongated tubular structure seen (red arrow) lateral to the right optic nerve extending from the superior rectus to the inferior rectus muscle

**Figure 3:**
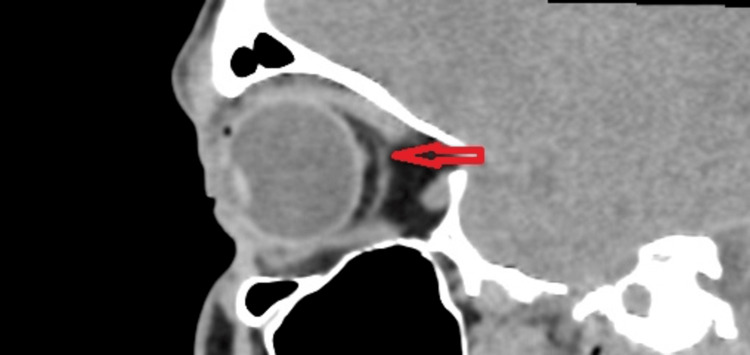
Saggital view of a plain computed tomography showing the orbit of the patient with an elongated tubular structure seen (red arrow) just behind the globe, extending from the superior to inferior rectus muscles

To investigate the possibility of extraocular muscle (EOM) involvement and to rule out space-occupying lesions, a plain CT scan of the brain and orbit was initially performed without contrast due to concerns about contrast-induced thyroid storm. The CT scan did not show any significant abnormalities except for a hyperintense tubular structure, resembling an anomalous vessel, observed between the superior and inferior rectus muscles, located just behind the right globe on the lateral aspect of the optic nerve and in close contact with the nerve (Figure 2: Coronal view of CT, Figure 3: Saggital view of CT).

**Figure 4:**
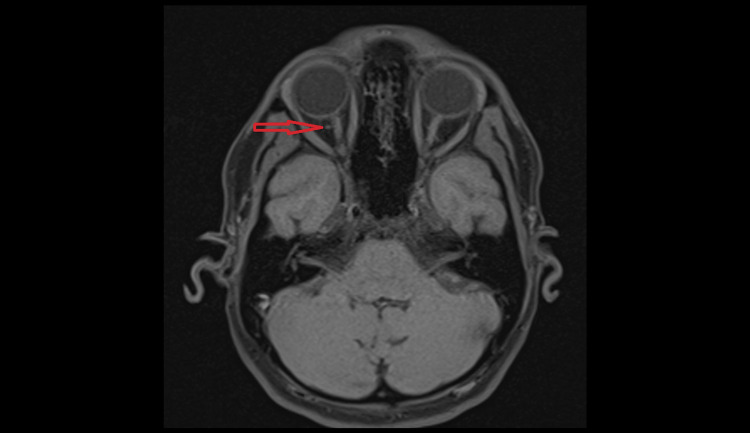
Axial view of a T1-weighted MRI pre-contrast showing an abnormal round structure (red arrow) located just behind the right globe and lateral to the optic nerve

**Figure 5:**
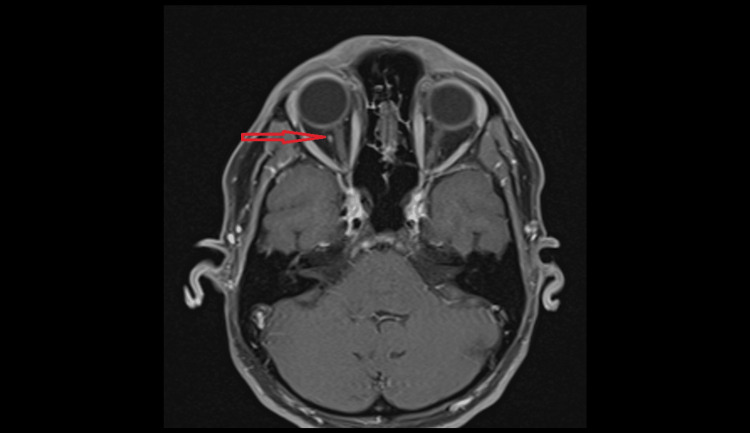
Axial view of a T1-weighted MRI post-contrast highlighting the same structure (red arrow) seen in Figure 4

**Figure 6:**
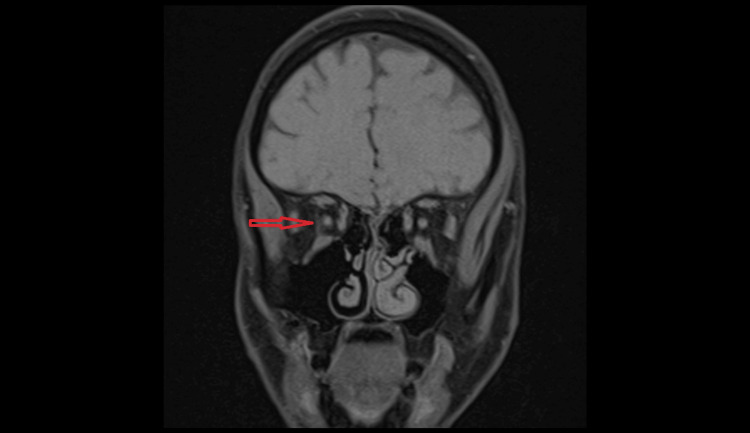
Coronal view of a T1-weighted MRI showing an abnormal, elongated, round structure (red arrow) running between the superior and inferior rectus muscle just lateral to the right optic nerve with a clear fat plane seen between both structures

A contrasted MRI of the brain and orbit was subsequently conducted to further evaluate the identified structure. The MRI confirmed the presence of the structure and showed signal characteristics that are analogous to those exhibited by EOMs (Figure 4: axial view of MRI pre-contrast, Figure 5: axial view of MRI post-contrast, Figure 6: coronal view of MRI). Importantly, no evidence suggestive of thyroid ophthalmopathy or space-occupying lesions was found in the MRI.

Based on the imaging findings, the patient was diagnosed with a SEOM, which is a rare anatomical variation. However, it was concluded that no active intervention was warranted for this patient. Instead, regular monitoring was recommended to ensure the stability of the condition and to promptly detect any structural changes in the SEOM if they occur in the future. There were no changes observed in the patient's symptoms or clinical signs post-initiation of carbimazole.

## Discussion

Several theories in the literature propose the development of these anomalous muscles. One hypothesis suggested by Whitnall in 1911 proposes that SEOMs may represent atavistic retractor bulbi muscles, typically found in reptiles, amphibians, and some ruminants but not in humans [4,5]. Another theory suggests an early disturbance in the development of the superior and inferior mesenchymal/mesectodermal complexes with incomplete division of some of its parts [6]. Khitri et al. also supported this theory, suggesting that the anomalous EOM bands may represent atrophied residual orbital remnants of aberrant muscular tissue that either were never innervated or lost innervation during development [3].

SEOMs may present incidentally, with strabismus, or with restriction in ocular motility. A case series involving 12 patients showed various presentations, including congenital fibrosis of EOM, Duane syndrome, vertical gaze palsy, infantile esotropia, partially accommodative esotropia, thyroid ophthalmopathy, and incidental findings [3]. Another case series with 12 patients reported that all cases had restrictive ocular motility, with horizontal and vertical deviations and reduced best-corrected visual acuity (BCVA) in affected eyes [2]. Most cases in this series demonstrated lid anomalies, particularly unequal lid aperture. Corrective strabismus surgery showed significant improvement in some cases. The reported cases involved an equal number of males and females, with bilateral involvement in only two cases, and the age range was 1.3 to 60 years old.

Attempts have been made to classify SEOMs in the literature. Lueder classified the muscles into three types based on their abnormal insertion points and courses [7]. The Demer classification is based on the direction of the involved recti [2]. Wang et al. proposed a four-type classification, including bilateral supernumerary EOMs, connections between EOMs, connections among EOMs with or without optic nerve involvement, and connections only between rectus muscles and the optic nerve [2].

In this case report, we present a patient with right-sided lid retraction and an asymmetrical palpebral aperture. Although the patient was diagnosed with hyperthyroidism, further investigation revealed no direct association between the thyroid status and ocular symptoms. However, imaging revealed an interesting finding that could be linked to the presenting symptoms. Upper eyelid retraction in thyroid eye disease can result from three distinct mechanisms: heightened sympathetic stimulation of Müller's muscle, excessive activity of the levator muscle as it contracts against a taut inferior rectus, or the formation of scar tissue between the levator and adjacent tissues. Nonetheless, given that the patient displayed no abnormalities in the extraocular muscles as revealed by imaging, these factors may not entirely account for the observed eyelid retraction [8]. However, considering the normal anatomical fibrous attachments between the superior rectus and levator, and factoring in the abnormal attachment of the SEOM to the superior rectus in this case, which may contribute to a certain amount of tractional force to the muscle itself, it may help explain the presence of the lid retraction as seen in our patient [9]. Additionally, it's crucial to emphasize that when assessing structures resembling an anomalous vessel running between the EOMs on imaging, future imaging studies should be conducted with heightened attention. This is particularly important now that we've established that SEOM variants can also manifest in this manner.

The key takeaway from this case highlights the potential complexities associated with a SEOM in a patient with thyroid eye disease. In a case study conducted by Baldeschi et al. involving a patient with Graves' ophthalmopathy and a swollen SEOM, a significant correlation was observed between the enlarged SEOM and the patient's strabismus [10]. The study suggests that similar to other EOMs, SEOMs can be influenced by thyroid ophthalmopathy, potentially exacerbating the clinical presentation. This discovery holds substantial clinical relevance for our patient, given the SEOM's proximity to the optic nerve and its connections to both the superior and inferior rectus muscles. Two noteworthy potential complications arise: first, the early possibility of optic nerve compression at the onset of thyroid ophthalmopathy, which could jeopardize the patient's vision; and second, the potential development of a more intricate and challenging form of strabismus due to muscle fibrosis, should it occur.

## Conclusions

In conclusion, this case report highlights a rare anatomical variation known as supernumerary extraocular muscles (SEOM), where anomalous extraocular muscles deviate from the normal muscle arrangement in the eye. While immediate intervention was not necessary for the SEOM, regular monitoring was advised to assess stability and detect any changes. Understanding potential associations between SEOM and conditions like thyroid ophthalmopathy and restrictive strabismus is crucial, as they may complicate diagnosis and management. This case report sheds light on the intriguing and diverse manifestations of SEOM within the eye, and how their presence can be associated with significant clinical implications, especially when coexisting with hyperthyroidism. However, due to its rarity and the limited knowledge surrounding SEOM, further research is needed to better comprehend its development and impact on ocular function.
